# Prognostic Classifier Based on Genome-Wide DNA Methylation Profiling in Well-Differentiated Thyroid Tumors

**DOI:** 10.1210/jc.2017-00881

**Published:** 2017-08-16

**Authors:** Mariana Bisarro dos Reis, Mateus Camargo Barros-Filho, Fábio Albuquerque Marchi, Caroline Moraes Beltrami, Hellen Kuasne, Clóvis Antônio Lopes Pinto, Srikant Ambatipudi, Zdenko Herceg, Luiz Paulo Kowalski, Silvia Regina Rogatto

**Affiliations:** 1 International Research Center, CIPE, A.C. Camargo Cancer Center and National Institute of Science and Technology in Oncogenomics, São Paulo 01509-010, SP, Brazil; 2 Department of Urology, Faculty of Medicine, UNESP, São Paulo State University, Botucatu 18618-970, SP, Brazil; 3 Department of Pathology, A.C. Camargo Cancer Center, São Paulo 01509-010, SP, Brazil; 4 Epigenetics Group; International Agency for Research on Cancer (IARC), Lyon 69372, France; 5 MRC Integrative Epidemiology Unit, University of Bristol, Bristol BS8 1TH, United Kingdom; 6 Department of Head and Neck Surgery and Otorhinolaryngology, A.C. Camargo Cancer Center, São Paulo 01509-010, SP, Brazil; 7 Department of Clinical Genetics, Vejle Hospital and Institute of Regional Health Research, University of Southern Denmark, Vejle, 7100, Denmark

## Abstract

**Context:**

Even though the majority of well-differentiated thyroid carcinoma (WDTC) is indolent, a number of cases display an aggressive behavior. Cumulative evidence suggests that the deregulation of DNA methylation has the potential to point out molecular markers associated with worse prognosis.

**Objective:**

To identify a prognostic epigenetic signature in thyroid cancer.

**Design:**

Genome-wide DNA methylation assays (450k platform, Illumina) were performed in a cohort of 50 nonneoplastic thyroid tissues (NTs), 17 benign thyroid lesions (BTLs), and 74 thyroid carcinomas (60 papillary, 8 follicular, 2 Hürthle cell, 1 poorly differentiated, and 3 anaplastic). A prognostic classifier for WDTC was developed *via* diagonal linear discriminant analysis. The results were compared with The Cancer Genome Atlas (TCGA) database.

**Results:**

A specific epigenetic profile was detected according to each histological subtype. BTLs and follicular carcinomas showed a greater number of methylated CpG in comparison with NTs, whereas hypomethylation was predominant in papillary and undifferentiated carcinomas. A prognostic classifier based on 21 DNA methylation probes was able to predict poor outcome in patients with WDTC (sensitivity 63%, specificity 92% for internal data; sensitivity 64%, specificity 88% for TCGA data). High-risk score based on the classifier was considered an independent factor of poor outcome (Cox regression, *P* < 0.001).

**Conclusions:**

The methylation profile of thyroid lesions exhibited a specific signature according to the histological subtype. A meaningful algorithm composed of 21 probes was capable of predicting the recurrence in WDTC.

## Teaser

An epigenetic signature of thyroid lesions is described based on the DNA methylation analysis. An algorithm was developed to predict recurrence, which has potential to be used in the clinical practice.

Thyroid cancer (TC) is the most common endocrine cancer (1) and comprises a group of tumors with remarkably different features. Papillary thyroid carcinoma (PTC) and follicular thyroid carcinoma (FTC) are well-differentiated thyroid carcinomas (WDTCs) comprising 90% of all TC cases, whereas poorly differentiated thyroid carcinoma (PDTC) and anaplastic thyroid carcinoma (ATC) subtypes are extremely aggressive and less frequently diagnosed (2). Despite the indolent biological behavior of the well-differentiated carcinomas, these tumors occasionally give rise to less differentiated and more aggressive TCs (3). Moreover, a significant number of patients with WDTC develop locoregional recurrences or even radioiodine-resistant distant metastases (3).

DNA methylation in TC has been intensively studied, and several markers have been described. To date, the majority of these reports are based on the analysis of candidate genes, focusing mainly in altered methylation in CpG islands and their association with gene expression (4, 5).

Currently, a limited number of genome-wide methylation studies have been reported in TC, and most of them are focused on PTC. Rodríguez-Rodero *et al.* (6), using a low-density platform (Illumina Infinium HM 27K microarray) in eight samples (two PTC, two FTC, two medullary, and two ATC), reported that different methylation profiles were associated with distinct TC subtypes. In 2014, The Cancer Genome Atlas (TCGA) project reported the methylation profiles in a large cohort of PTC cases (n = 496) performed in a high-density platform (Illumina Infinium HM 450 array) (7). Blending different genomic approaches, the authors proposed a PTC classification based on the molecular subtypes and recognized potentially useful candidates to contribute to management of the disease.

Aberrant DNA methylation has emerged as an important mechanism involved in cancer progression. In PTC samples, the methylation pattern of *TIMP3, SLC5A8,* and *DAPK* was significantly associated with extrathyroidal invasion, lymph node metastasis, multifocality, advanced tumor stages, and *BRAF* mutation (8). In 2014, Wang *et al.* (9) showed that *RUNX3* site-specific hypermethylation significantly increased the risk of PTC recurrence. Using genome-wide methylation profiling (Illumina Infinium HM 27K platform) in well-differentiated thyroid tumors and validating the data in an independent series of cases, Mancikova *et al.* (10) identified *WT1* and *EI24* as markers for the risk of developing tumor recurrence. Overall, these findings indicate that methylation and prognosis have been poorly explored in TCs.

In this study, a comprehensive global methylation (Illumina Infinium HM 450K microarray) analysis was performed in a large cohort of cases including nonneoplastic thyroid tissues (NTs), benign thyroid lesions (BTLs), and malignant thyroid lesions. Biomarkers and biological pathways epigenetically regulated involved in thyroid carcinogenesis were described. Subsequently, we developed a prognostic classifier capable of predicting the recurrence in patients with WDTC. Performance of the DNA methylation-based algorithm was tested against the TCGA database.

## Materials and Methods

### Patients

The samples were retrospectively obtained from patients who underwent surgery at A.C. Camargo Cancer Center, São Paulo, Brazil. The study was approved by the Institutional Ethics Committee (protocol no. 475.385). The patients were advised of the procedures and provided written informed consent. All FTC/Hürthle cell carcinoma (HCC) and PDTC/ATC samples available in the institutional tumor biobank were included, whereas the 60 PTC samples were selected from patients treated with total thyroidectomy followed by radioiodine therapy (to standardize the treatment) and with ≥5 years of follow-up. Ten FTC/HCC, four PDTC/ATC, eight follicular adenoma (FA), six nodular goiter, three lymphocytic thyroiditis (LT), and 50 surrounding normal tissue samples from patients with PTC were also included in this study. A subset of samples (41 PTCs matched with adjacent NTs) used in this analysis was previously reported (11). Clinical and histopathological data for the malignant lesions are summarized in the Table 1.

**Table 1.** **Clinicopathologic Characteristics of the Patients Enrolled in the Study**

Characteristics	PTC*^a^*		Other Subtypes	
	n	%	n	%
Age				
<55 y	51	85	4	28.6
≥55 y	9	15	10	71.4
Sex				
Female	44	73.3	10	71.4
Male	16	26.7	4	28.6
Histology (excluded PTC)				
FTC	—	—	8	57.1
HCC	—	—	2	14.3
PDTC	—	—	1	7.1
ATC	—	—	3	21.4
Predominant variant (PTC only)				
Classic	47	78.3	—	—
Follicular	10	16.7	—	—
Other	3	5.0	—	—
Tumor dimension, cm				
Median (interval)	1.3	—	2.3	—
	(0.5–5.5)		(0.9–13)	
Micro-PTC (≤1 cm)	25	41.7	—	—
PTC (>1 cm)	35	58.3	—	—
Extrathyroidal extension				
No	30	52.6	7	53.8
Yes	27	47.4	6	46.2
Ni	3	—	1	—
Invasion				
Vascular	2	3.4	5	38.5
Lymphatic	3	5.1	1	7.7
Both	1	1.7	2	15.4
No	53	89.8	5	38.5
Ni	1	—	1	—
Perineural invasion				
No	42	93.3	10	23.1
Yes	3	6.7	3	76.9
Ni	15		1	—
Lymph node metastasis				
No (cN0, pN0)	32	53.3	9	69.2
Yes (pN1)	28	46.7	4	30.8
Ni	—	—	1	—
Clinical evolution				
Free of disease	44	73.3	5	71.4
Confirmed recurrence	16	26.7	2	28.6
Ni*^b^*	—	—	7	—
Death				
No	59	98.3	10	71.4
Yes	1	1.7	4	28.6
Follow-up (mo)*^c^*	83.8	—	63.3*^d^*	—
	(1.1–142.9)		(4.0–139.4)	

cN0, no clinical evidence of cancer in the regional lymph nodes; Ni, not informed; pN0, no pathological evidence of cancer in regional lymph nodes; pN1, pathological confirmation of cancer in regional lymph nodes.

^a^ PTC samples were enriched by more aggressive features and recurrence in follow-up as a consequence of the inclusion criteria (only patients submitted to total thyroidectomy followed by radioiodine therapy and patients without recurrence with ≥5 years of follow-up; all relapsed patients were enrolled).

^b^ Three FTCs had <5 years of follow-up with no recurrence, and 4 undifferentiated carcinomas showed <6 months of follow-up after the diagnosis.

^c^ Data up to August 2014.

^d^ Only for FTC samples.

### DNA extraction, bisulfite conversion, and DNA methylation analysis

Genomic DNA from fresh frozen thyroid tissues was extracted *via* the phenol-chloroform method and quantified by Qubit® dsDNA BR Assay (Qubit® 2.0 Fluorometer, Life Technologies, Carlsbad, CA). The DNA (500 ng) was bisulfite converted with an EZ DNA Methylation-Gold™ Kit (Zymo Research, Irvine, CA) according to the manufacturer’s recommendations.

Genome-wide methylation assays were performed with the Human Methylation 450 BeadChip (Illumina, San Diego, CA). The data were captured in Illumina HiScan system, and the *β* values ranged from 0 (*unmethylated*) to 1 (*methylated*). R language was used for the methylation analysis, as previously described (12). The quality control parameters were followed, probes were filtered and normalized, and the batch effects were assessed. Briefly, cross-reactive probes (≥49 bases), single nucleotide polymorphisms (minor allele frequency >5%), sex-associated probes (13), low-quality probes (*P* > 0.05), and low bead count (<3) in ≥5% of samples were excluded with the WaterRmelon package. Data were normalized *via* the beta-mixture quantile normalization method (14). The sva package was used to correct the batch effects (15). The annotation was performed according to the Illumina (hg19) and HUGO Gene Nomenclature Committee.

Unsupervised hierarchical clustering analysis was performed as described in Supplemental Material with BRB Array Tools version 4.4.0 software (Biometric Research Branch, National Cancer Institute). Differentially methylated loci were identified by comparing BTL, PTC, FTC/HTC, and PDTC/ATC samples with NT by using the limma package, adopting an adjusted *P* < 0.05 and mean delta beta (*Δβ*) <−0.2 or >+0.2. Raw microarray and normalized data are available in Gene Expression Omnibus (http://www.ncbi.nlm.nih.gov/geo/) under specific accession number (GSE97466).

### Pathway enrichment analysis

*In silico* pathway analysis was conducted with Ingenuity Pathway Analysis (IPA®, QIAGEN, Redwood City, CA, www.qiagen.com/ingenuity) and KOBAS (version 2.0; http://kobas.cbi.pku.edu.cn/home.do) software, comprising the list of differentially methylated probes obtained in all comparisons (BTL *vs* NT, PTC *vs* NT, FTC/HCC *vs* NT, and PDTC/ATC *vs* NT). Only probes mapped within the interval until 200 bp and 200 to 1500 bp upstream of the transcription start site and those in CpGs located within the 5′UTR and in the first exon were included.

### DNA methylation-based algorithm to predict well-differentiated (PTC and FTC/HCC) TC outcomes

Differentially methylated probes with potential biological significance in more aggressive subtypes of thyroid malignancies were initially selected to design a prognostic classifier. This first step consisted in obtaining probes located in the promoter regions by comparing between PDTC/ATC and NT. WDTC (PTC and FTC/HCC) was grouped in poor prognosis (PP) and good prognosis (GP) cases. Patients with confirmed recurrent locoregional disease (histological analysis) or distant metastasis (conclusive imaging test) in the follow-up were categorized as having WDTC-PP. The WDTC-GP group included only patients without any suspicion of active disease on clinical follow-up (normal serum thyroglobulin levels and no evidence of disease in the imaging screening) for ≥5 years (WDTC-GP: 43 PTC and 5 FTC). Because multifocal thyroid tumors are probably related to an independent clonal origin (16) and to obtain a more robust analysis, WDTC-PP exhibiting multifocal primary tumors was excluded (WDTC-PP: 6 PTC and 2 FTC).

The *Δβ* >|0.1| was used as a criterion for the comparison between WDTC-PP, NT, and BTL, which allowed the filtering of probes exclusively altered in WDTC-PP and ATC/PDTC. The remaining probes were submitted to a diagonal linear discriminant analysis (DLDA) method. The classification performance was calculated with a leave-one-out cross-validation test (BRB Array Tools version 4.4.0). The predictive model used was based on scores stratified as low (NT range), intermediate (below DLDA threshold), and high (above DLDA threshold).

### Application of the prognostic classifier in PTC samples from the TCGA database

TCGA database (http://tcga-data.nci.nih.gov/tcga/) was used to test the performance of the DNA methylation-based algorithm. Methylation (level 3, 450k Illumina platform; November 2015) and clinical data were obtained from TC samples, which comprised only patients with PTC. Clinical data were thoroughly evaluated under the same inclusion criteria previously defined in our study, resulting in 48 WDTC-GP and 14 WDTC-PP. The *in silico* data were used to test performance of the mathematical model previously established without any further adjustment.

### Data processing and statistical analysis

Methylation quality control data and differential analysis were performed in R and Bioconductor packages (methylumi, sva, limma). The Bonferroni method was used to adjust *P* values for multiple comparisons. DNA methylation *β*-values were graphically represented in a heatmap generated by BRB ArrayTools. Disease-free survival analysis (SPSS version 21.0; Chicago, IL) was performed *via* Kaplan–Meier estimator with log‐rank test and multivariable analysis with Cox (proportional hazards) regression, adopting a two-tailed *P* < 0.05 value as significant.

## Results

### Global DNA methylation patterns unveils TC subtypes

**Figure 1.**
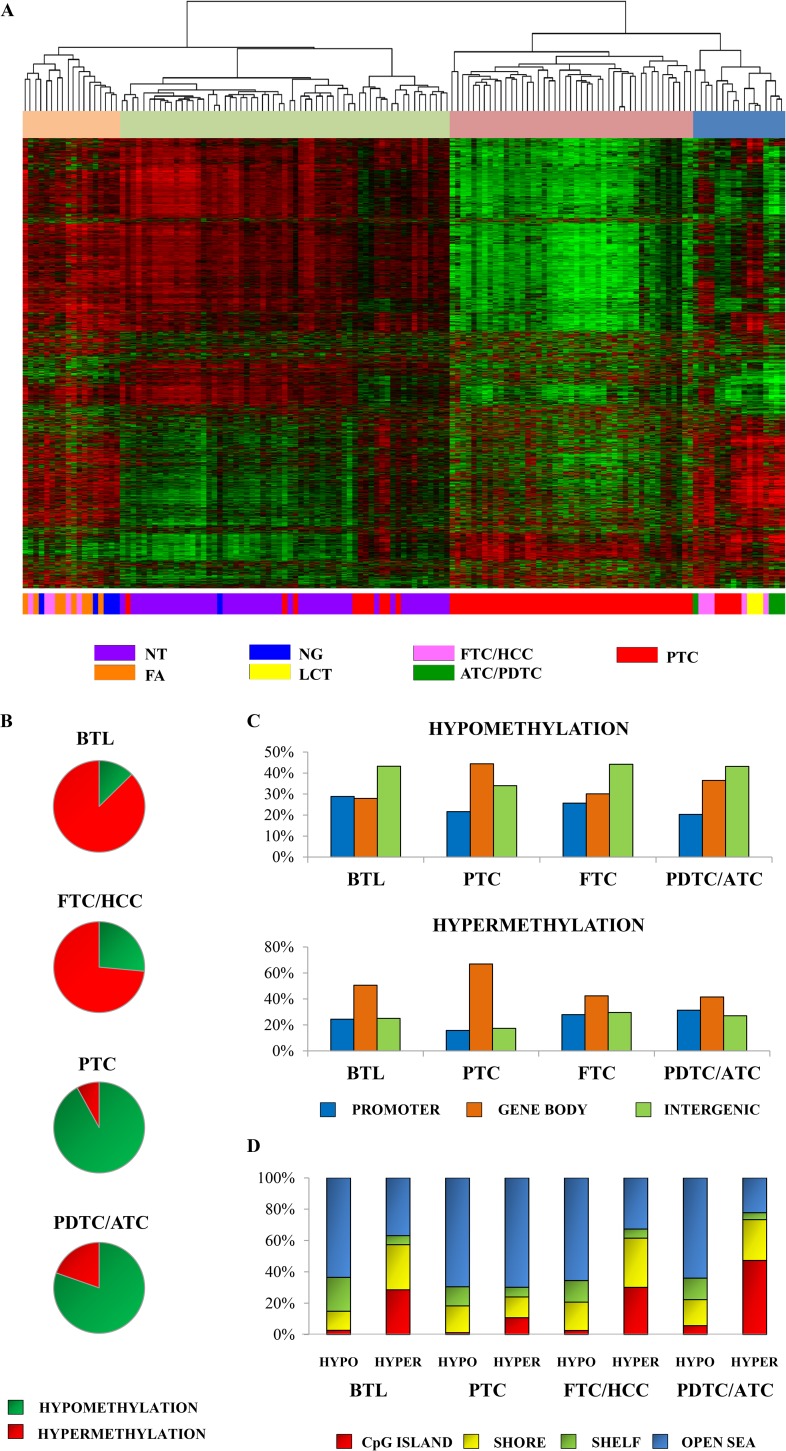
Comparison of CpG site methylation regions between benign lesions, TCs, and surrounding NTs. (A) Unsupervised hierarchical clustering analysis of the methylation profiles of thyroid tissues using the most variable probes within the platform (8016 probes). Four major groups were detected and defined by the most variable probes. The rows indicate the CpG sites, and the columns indicate the samples. (B) Percentage of hypermethylated (red) and hypomethylated (green) probes in BTL, PTC, FTC/HCC, and PDTC/ATC. (C) CpG site proportions by location relative to promoter, gene body, and intergenic region, according to Illumina 450 K annotation. (D) CpG site proportions by location relative to CpG island regions, according to Illumina 450 K annotation.

DNA methylation profiles of 74 tumor samples, 50 surrounding NTs, and 17 BTLs were investigated. Unsupervised hierarchical clustering analysis revealed four clusters with pathological and molecular differences (Fig. 1A): cluster 1 was enriched with FAs, nodular goiters, and minimally invasive FTCs, showing more frequent hypermethylation events; cluster 2 displayed all NT samples; cluster 3 comprised exclusively PTC samples, characterized by an evident methylation loss; and cluster 4 exhibited all ATC/PDTC, lymphocytic chronic thyroiditis, and the remaining FTC/HCC (most extensively invasive). In contrast with the normal-like cluster (cluster 2), cluster 4 was enriched by alterations involved in immune response genes and clusters 1 and 3 by signal transduction–related genes (Supplemental Table 1).

The comparison of BLT, PTC, FTC/HCC, and PDTC/ATC *vs* NT (|*Δβ*| > 0.2 and adjusted *P* < 0.05) revealed 1753 differentially methylated probes in BTL (222 hypomethylated and 1531 hypermethylated), 3015 in PTC (2773 hypomethylated and 242 hypermethylated), 5575 in FTC/HCC (1475 hypomethylated and 4100 hypermethylated), and 35,167 in PDTC/ATC (28,252 hypomethylated and 6195 hypermethylated) (Fig. 1B; Supplemental Table 2).

DNA methylation changes were found in all genomic regions. The follicular lesions presented hypomethylated and hypermethylated CpG sites closer to the transcription start sites in gene body and intergenic regions (Fig. 1C). Hypermethylation of CpG island regions was identified in all comparisons, whereas hypomethylation was more frequently observed outside the CpG islands (Fig. 1D). A total of 620 hypermethylated probes was exclusively found in BTL, 113 in PTC, 2244 in FTC/HCC, and 5411 in PDTC/ATC. Similarly, 95 hypomethylated probes were found in BTL, 1,996 in PTC, 465 in FTC/HCC, and 26,734 in PDTC/ATC (Supplemental Fig. 1).

### Molecular pathways associated with differentially methylated promoter regions

*In silico* analysis using the list of promoter regions hypomethylated and hypermethylated obtained in the comparison between thyroid lesions and NTs revealed the involvement of different pathways according to the tumor subtype (Table 2; Supplemental Table 3). Activation of vitamin D receptors (VDRs) and retinoid X receptors (RXRs) was associated with FTC and PTC (IPA *P* < 0.05), whereas the G-protein alpha-i (*Gαi*) signaling pathway was identified as deregulated in ATC/PDTC (Supplemental Fig. 2, Supplemental Table 3) (adjusted *P* < 0.05).

**Table 2.** **Top 10 Significant Canonical Pathways Identified in the Comparisons by Ingenuity Pathway Analysis Software**

Analysis	Pathway (Ingenuity Pathway Analysis)	Prediction State (*z* Score)	Genes Found/Database	*P* (Fisher Exact Test)	*P* (Benjamini–Hochberg Correction)
BTL *vs* NT	Reelin signaling in neurons	—	6/79	0.002	0.438
	eNOS signaling	Inhibited (−1.9)	8/142	0.003	0.438
	Leukocyte extravasation signaling	Inhibited (−0.7)	9/198	0.006	0.443
	IL-4 signaling	—	5/76	0.009	0.443
	CCR3 signaling in eosinophils	—	6/117	0.014	0.443
	FAK signaling	—	5/87	0.015	0.443
	l-dopachrome biosynthesis	—	1/1	0.017	0.443
	RhoA signaling	Inhibited (−2.2)	6/122	0.017	0.443
	Amyotrophic lateral sclerosis signaling	—	5/98	0.025	0.443
	IL-12 signaling and production in macrophages	—	6/135	0.026	0.443
FTC/HCC *vs* NT	Inhibition of matrix metalloproteases	—	6/39	0.014	0.946
	Leukocyte extravasation signaling	Inhibited (−1.4)	18/198	0.015	0.946
	eNOS Signaling	Inhibited (−1.7)	14/142	0.016	0.946
	Tec kinase signaling	Inhibited (−1.9)	15/158	0.017	0.946
	HGF signaling	Inhibited (−2.1)	11/105	0.020	0.946
	Reelin signaling in neurons	—	9/79	0.021	0.946
	FAK signaling	—	9/87	0.036	0.946
	Fatty acid *α*-oxidation	—	3/16	0.047	0.946
	VDR/RXR activation	Activated (0.4)	8/78	0.049	0.946
	Factors promoting cardiogenesis in vertebrates	—	9/92	0.049	0.946
PTC *vs* NT	TREM1 signaling	Activated (2.8)	8/74	<0.001	0.044
	IL-10 signaling	—	7/68	<0.001	0.079
	RhoGDI signaling	Inhibited (−2.3)	10/173	0.003	0.192
	Signaling by Rho family GTPases	Activated (2.1)	12/234	0.003	0.192
	The visual cycle	—	3/15	0.003	0.192
	LXR/RXR activation	Inhibited (−2.1)	8/121	0.003	0.192
	VDR/RXR activation	Activated (2.2)	6/78	0.005	0.232
	LPS/IL-1 mediated inhibition of RXR function	Activated (1.0)	11/219	0.005	0.232
	Integrin signaling	Activated (3.0)	10/201	0.008	0.301
	Role of JAK1 and JAK3 in *γ*c cytokine signaling	—	5/63	0.009	0.301
PDTC/ATC *vs* NT	Agranulocyte adhesion and diapedesis	—	58/189	<0.001	0.001
	Atherosclerosis signaling	—	41/124	<0.001	0.002
	LXR/RXR activation	Inhibited (−1.0)	39/121	<0.001	0.004
	Transcriptional regulatory network in embryonic stem cells	—	18/40	<0.001	0.004
	FXR/RXR activation	—	39/127	<0.001	0.009
	Granulocyte adhesion and diapedesis	—	50/177	<0.001	0.009
	GABA receptor signaling	—	23/67	<0.001	0.031
	Estrogen biosynthesis	—	15/37	<0.001	0.033
	*Gαi* signaling	Activated (2.8)	35/120	<0.001	0.033
	*Gαs* signaling	Activated (1.0)	32/109	<0.001	0.045

### Prognostic classifier performance in well-differentiated TC

**Figure 2.**
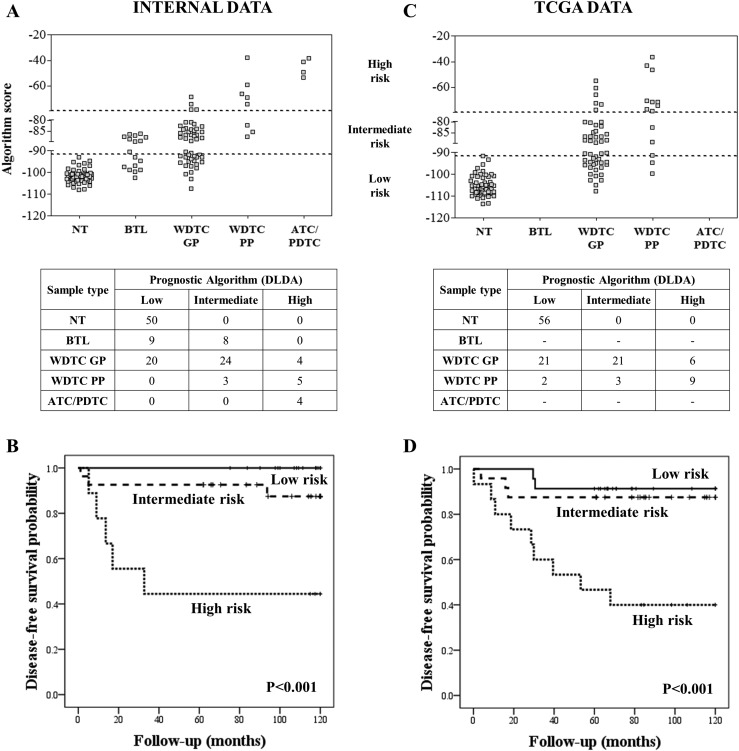
Prognostic evaluation based on the methylation data. (A, C) Predictive model values based on the 21 probes used in the stratification of the cases in three categories: low, intermediate, and high risk using both internal and external data (TCGA). (B, D) Disease-free survival curves in well-differentiated carcinomas stratified into low, intermediate, and high risk according to the classifier in both internal and external data (TCGA).

An altered DNA methylation pattern in a specific locus has the potential to be a useful biomarker for the identification of aggressive TC subtypes. Based on this premise, a prognostic classifier was designed with 7700 probes mapped in the promoter regions obtained from the comparison between PDTC/ATC and NT. A total of 21 probes showed Δ*β* <−0.1 or >+0.1 comparing WDTC-PP with WDTC-GP cases, with 17 hypomethylated (*OR6K2, LAIR2, PFKFB2, OR4K15, NLRP11, OR9G4, THSD7B, OR2M3, OR52B2, OR2T6, FFAR2, TN3, OR52M1, DCD, ADGRE2, HRH1,* and *CXXC5*) and four hypermethylated (*GPR21, MBP, YPEL4,* and *ATP6V0C*) in the WDTC-PP group (Table 3). These 21 probes were submitted to a DLDA to train a prognostic predictor classifier, which was able to distinguish WDTC-PP from WDTC-GP with 63% sensitivity and 92% specificity (63% sensitivity and 90% specificity in leave-one-out cross-validation). Performance according to stratification of the predictive model in low, intermediate, and high scores is shown in Fig. 2A.

**Table 3.** **The Prognostic Classifier Comprising 21 Probes Included 4 Hypermethylated (DLDA Positive Values) and 17 Hypomethylated (DLDA Negative Values) Probes**

Probe ID	Gene Symbol	Promoter Region	*Δβ* ATC/PDTC–NT	*Δβ* WDTC-PP–WDTC–GP	DLDA Score
cg00119186	*OR6K2*	1st exon	−0.411	−0.105	−16.72
cg08905487	*LAIR2*	TSS200	−0.406	−0.134	−12.14
cg02710090	*PFKFB2*	TSS1500	−0.253	−0.109	−11.41
cg07531287	*OR4K15*	1st exon	−0.280	−0.112	−10.68
cg25730098	*NLRP11*	5′UTR	−0.321	−0.118	−10.65
cg03178489	*OR9G4*	1st exon	−0.307	−0.104	−9.33
cg08231096	*THSD7B*	TSS200	−0.352	−0.101	−9.23
cg07598464	*OR2M3*	1st exon	−0.388	−0.131	−8.41
cg21966764	*OR52B2*	TSS1500	−0.263	−0.110	−7.31
cg06846214	*OR2T6*	TSS1500	−0.337	−0.109	−6.38
cg20199836	*FFAR2*	TSS200	−0.369	−0.102	−6.06
cg17372806	*RTN3*	TSS1500	−0.330	−0.103	−5.81
cg03316101	*OR52M1*	1st exon	−0.309	−0.123	−5.03
cg01687040	*DCD*	TSS1500	−0.355	−0.103	−4.75
cg04103514	*ADGRE2*	TSS200	−0.379	−0.122	−4.70
cg06457736	*HRH1*	TSS200	−0.460	−0.120	−3.48
cg19628988	*CXXC5*	5′UTR	−0.362	−0.104	−3.28
cg14115756	*GPR21*	TSS1500	0.457	0.117	4.07
cg03560685	*MBP*	TSS1500	0.513	0.109	4.98
cg22705929	*YPEL4*	TSS1500	0.324	0.122	5.66
cg05884711	*ATP6V0C*	TSS1500	0.341	0.191	9.60

Disease-free survival analysis revealed a higher risk of recurrence according to the score categories (Fig. 2B). Multivariable analysis showed that high score is an independent factor for lower disease-free survival (*P* < 0.001; odds ratio 31.0; 95% CI, 4.9 to 195.6) (Supplemental Table 4). The prognostic classifier found in our analysis was tested with the TCGA methylation data in PTC samples, confirming a similar predictive accuracy (64% sensitivity and 88% specificity) (Fig. 2C) and lower disease-free survival in cases with high scores (Fig. 2D). Contrasting the methylation definition of “high risk” with the clinical–pathological “high risk” classification (17), the latter presented lower sensitivity (21% to 38%) and slightly higher specificity (96% to 98%) (Supplemental Fig. 3). The individual clinical pathological data and algorithm scores of internal and external (TCGA) data sets are described in Supplemental Table 5.

## Discussion

The thyroid samples derived from follicular cells revealed high methylation variability, as showed in the unsupervised hierarchical clustering analysis. From the four major groups exposed as results of this analysis, LT was grouped with highly aggressive tumors including undifferentiated or poorly differentiated tumors and extensively invasive FTCs (cluster 4). Lymphocytic thyroiditis is a chronic autoimmune thyroid disease enriched with immune cell infiltration and inflammatory cytokines (18, 19), similar to highly proliferative and invasive thyroid tumors (20). Interestingly, an enrichment of genes related to immunological response was detected in cluster 4. Therefore, an important source of the methylation heterogeneity may be the inflammation response, as previously described in molecular profiling studies of thyroid tumor samples (21, 22).

The methylation profiles of the sample groups compared with NTs revealed that CpG site hypermethylation was more frequently observed in benign lesions and FTCs and hypomethylation in papillary and poorly differentiated or anaplastic carcinomas. These findings point out distinct epigenetic mechanisms related to the development or progression of thyroid lesions according to histological subtype.

Changes in the methylation pattern of benign lesions have also been reported (23), suggesting the relevance of this process at early stages of tumorigenesis. CpGs with methylation gains were more frequently observed in FTCs (4100) than in BTLs (1531) compared with NTs. Mancikova *et al.* (10) reported similar findings comparing gene promoter methylation profiles of FTCs and FAs. We also found aberrant methylation in gene body in both lesions, BTL and FTC. In addition to the morphological and genetic similarities described in adenomas (in our analysis, eight FAs were included in the BTL group) and FTCs (24), these entities also shared an elevated number of hypermethylated CpGs (479) (Supplemental Fig. 1).

PTCs displayed fewer aberrantly methylated genes compared with other carcinoma subtypes. In accordance with previous studies using methylation arrays in PTC samples, loss of methylation in both promoter and gene body regions was more frequently observed than hypermethylation (10, 25). Moreover, a high degree of loss of methylation was found in more aggressive undifferentiated TCs, a similar finding previously described in acute myeloid leukemia and in breast cancer progression (26, 27). All histological subtypes of thyroid lesions exhibited hypermethylation in CpG island promoters as frequent event; hypomethylation in CpGs outside the promoter regions was also detected. These findings are in agreement with global methylation studies performed in TC (6, 10).

*In silico* pathway analysis *via* probes mapped in the promoter region revealed the activation of the *Gαi* pathway in poorly differentiated and undifferentiated carcinomas. These G proteins stimulate the kinase activity of cSRC protein (28), which is involved in a variety of cell functions, including survival, proliferation, and cell migration. In our study, *Gαi,* *SHC,* and *RAS* were hypomethylated, and *RAP1GAP* (negative regulator of *Gαi* pathway) was hypermethylated. Using gene expression analysis, we previously reported *RAP1GAP* down-expression in PTC samples (21). In thyroid cell lines, *RAP1GAP* downregulation was associated with migratory and invasive properties (29). Moreover, *RAP1GAP* promoter hypermethylation and downregulation were reported in the most aggressive forms of TC (30). Our data give additional support to the involvement of the SRC upstream regulators in the prognosis of poorly differentiated and undifferentiated TCs.

Previously, we detected the VDR and RXR activation pathway as potentially deregulated in PTC samples (21), which is probably related to DNA methylation changes. In the current study, we demonstrated the predicted activation of this pathway in FTC samples but not in PDTC and ATC. These results are in agreement with high expression levels of VDR (31) and RXR (32) described in differentiated TC compared with normal tissues. Furthermore, high expression levels of VDR in patients with ATC are considered rare (31). These results reinforce that methylation is an important mechanism of gene regulation related to tumor progression, because of the importance of VDRs and RXRs in growth and cell differentiation.

Studies aiming to identify reliable markers associated with TC aggressiveness at the time of diagnosis can result in individualized treatment. Tumors harboring *BRAF* mutations have been associated with more aggressive phenotypes (3). Recently, *TERT* promoter mutations were associated with a worse prognosis, especially in *BRAF* mutated cases. Approximately 9% of PTCs were positive for *TERT* mutations (particularly C228T and C250T), whereas PDTCs and ATCs showed a higher frequency of mutations (40% and >70%, respectively) (33).

Methylation analysis has been described as a potent strategy to predict outcomes in several cancer subtypes. In PTCs, *RUNX3* hypermethylation (9) and *TSHR* hypomethylation (34) were reported as related to tumor recurrence. We found *RUNX3* hypermethylated in the promoter region (13 probes) in PDTC/ATC, but no association with recurrence was observed. Nonetheless, one probe (cg00117172) mapped in the nonpromoter region of *RUNX3* (body gene) was hypermethylated in both comparisons, PDTC/ATC *vs* NT (*Δβ* = 0.313) and WDTC-PP *vs* WDTC-GP (*Δβ* = 0.102), reinforcing their potential as biomarkers of tumor aggressiveness.

In this study, using rigorous inclusion criteria aiming to minimize confounding factors, we described a DNA methylation model to predict WDTC outcome, in terms of recurrence. The model initially was designed based on the assumption that promoter regions differentially methylated in anaplastic and poorly differentiated tumors, which by definition present PP, could also be involved in the more aggressive forms of well-differentiated carcinomas. After filtering the initial list by comparing WDTC-PP with WDTC-GP, NT, and BTL, we designed a mathematical model based on 21 probes. Considering that LT and highly aggressive tumors presented similar methylation profiling, which may be explained by the strong influence of inflammation, the comparison of WDTC-PP with BTL was an essential step to avoid the prediction of inflammatory lesions as high-risk cases. In our cases, no significant differences were found considering the LT in the groups predicted by the DNA methylation prognostic model (low, 30%; intermediate, 19%; high, 33%; Fisher exact test *P* = 0.541).

From the probes included in the algorithm, 17 of 21 showed loss of methylation. Interestingly, 7 of 17 probes are mapped in the promoter regions of olfactory receptor genes. Olfactory receptors are G protein–coupled receptors, which have been reported as mediating tumor proliferation, invasiveness, and metastasis (35, 36). Although these genes are included among those described in the algorithm, their role in TC prognosis remains to be clarified.

Interestingly, high expression of *PFKFB2,* a hypomethylated gene found in the classifier, was reported as being associated with advanced TNM stage and also was implied in poor overall survival in hepatocellular carcinoma (37). In TC, *PFKFB2* overexpression was described in older patients (>40 years old) and associated with PP (38).

The methylation signature of 21 probes herein introduced was capable of discriminating 86% of the samples according to the outcome, demonstrating high specificity (90%) but lower sensitivity (63%). Therefore, more than half of WDTC-PP cases would be detected, with no further implications for overtreatment, which is a well-discussed issue in the management of patients with WDTC (39). A similar predictive power was observed when the algorithm was tested in an independent *in silico* data set of PTC samples (TCGA), which correctly classified 82% of the samples (64% sensitivity and 88% specificity).

Performance of the methylation algorithm was compared with *TERT* promoter mutation (C228T and C250T) with the TCGA data (data not shown). Fifty of the 62 PTC samples included in the TCGA cohort presented *TERT* promoter sequences. Four of these 50 cases were positive for the mutation: Two were classified by the methylation algorithm as intermediate (all disease-free) and two as high risk (one disease-free and one relapsed case). In addition, the mutation assay was unable to identify relapsed patients with TC (8 of 9). Therefore, the methylation algorithm and *TERT* promoter mutation analysis showed no overlapping. The performance of our methylation algorithm was also compared with the American Thyroid Association guidelines (17), revealing a distinct set of high-risk cases. The high risk prediction based on molecular or clinical–pathological methods increased the sensitivity to 75% and specificity to 90% in the internal data set and 71% sensitivity and 88% specificity in the TCGA data set. These findings suggested that the methylation test would be feasible in clinical routines in combination with conventional stratification methods. A multiplexed methylation assay could be used to increase its applicability, similar to other tools described in the literature (40).

## Conclusions

Thyroid carcinoma subtypes, which present the same cellular origin, showed different DNA methylation profiling. A prognostic classifier based on the methylation changes in a small number of loci was reported, which could correctly classify a subset of patients with WDTC with poor outcomes. Even though our study and the TCGA data revealed a limited number of cases classified as WDTC-PP, the rigorous criteria used to select the patients and the power to predict recurrence in both analyses reinforce that this classifier has the potential to be applied as a prognostic tool useful for making therapeutic decisions in TC.
