# Supplementary material for: Prognostic Classifier Based on Genome-Wide DNA Methylation Profiling in Well-Differentiated Thyroid Tumors
Source: J Clin Endocrinol Metab. 2017 Aug 16;102(11):4089–99. doi: 10.1210/jc.2017-00881 (PMC5673278; doi:10.1210/jc.2017-00881)
Supplement: Supplementary file 1 [file jc.2017-00881.sm1.docx]

**Supplemental Material**

**Sample selection decision**

Patient samples were included in this retrospective study according to the availability of A.C. Camargo Cancer Center BioBank. We included 60 papillary carcinomas (PTC), 10 follicular/Hürthle cell carcinomas (FTC/HCC), four poorly differentiated/anaplastic carcinomas (PDTC/ATC), eight follicular adenomas (FA), six nodular goiters (NG), three lymphocytic thyroiditis (LT) and 50 surrounding normal tissues from PTC patients (NT). Two FTC and one PTC samples were excluded from clinical data comparisons for loss of follow-up.

**Unsupervised clustering analysis**

Unsupervised hierarchical clustering analysis was performed using complete linkage and one-minus-correlation distance parameters, comprising the most variable methylated probes (interquartile range >0.2) with BRB Array Tools v. 4.4.0 software (Biometric Research Branch, National Cancer Institute). The clusters comparison was performed using multi-class test in BRB Array Tools (P adjusted< 0.001). A Δβ > |0.2| between the tumor enriched (cluster 1, 3 and 4) and the normal-like (cluster 2) clusters was established. The *in silico* pathway analysis was carried out including probes covering the promoter regions using KOBAS tool (v2.0).

**
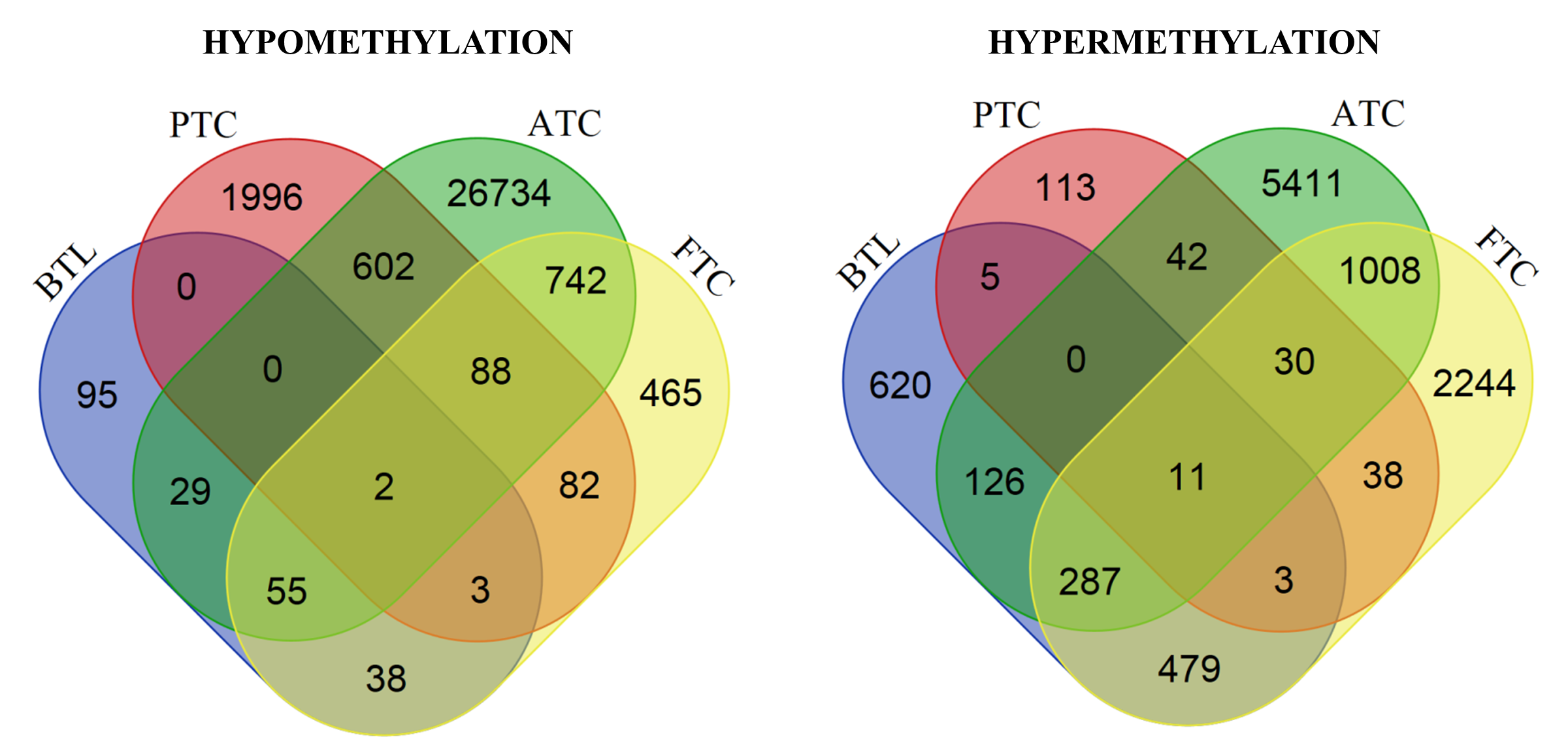
**

**Supplemental Figure 1.** Comparison of CpG site methylation regions among benign lesions, thyroid carcinomas and surrounding nonmalignant tissues showing hyper-and hypomethylation according to the pathological subtypes revealed common and exclusive alterations.

**
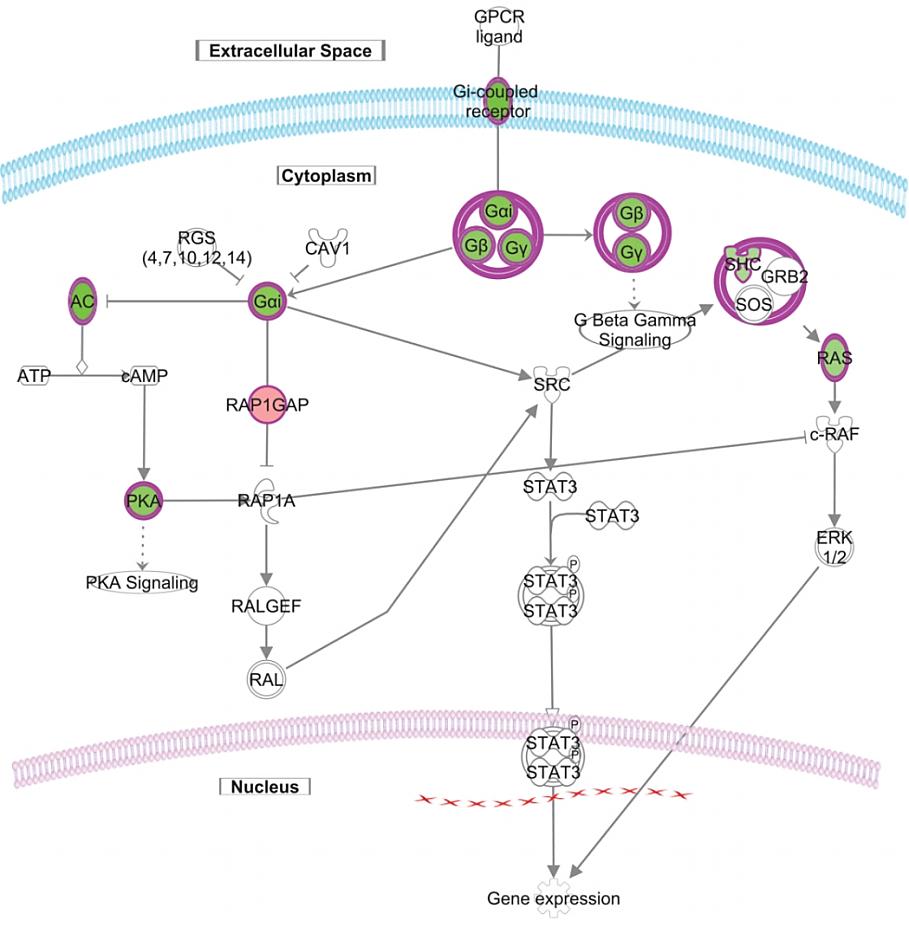
Supplemental Figure 2.** Gαi signaling canonical pathway was detected as involved in poorly differentiated thyroid/anaplastic carcinoma (IPA software). By comparing ATC/PDTC with NT, hypomethylation in many members of this pathway could be noticed, except for *RAP1GAP* (hypermethylated). The activation of the pathway was predicted by IPA software after conversion of hypomethylated and hypermethylated genes into putative overexpressed and underexpressed genes, respectively. Green: hypomethylation; Red: hypermethylation in ATC/PDTC compared to non-neoplastic thyroid tissues.

**
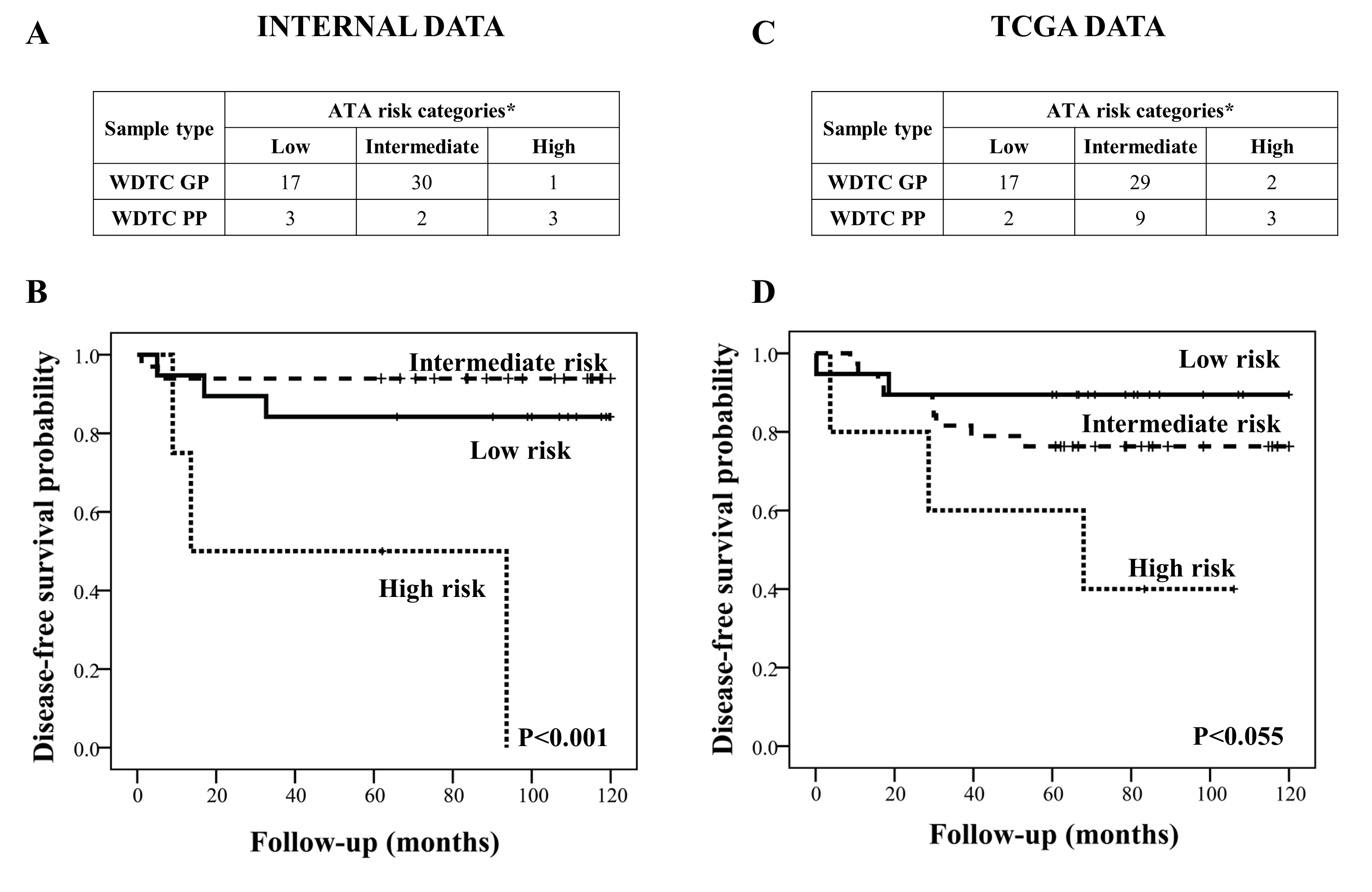
Supplemental Figure 3:** Recurrence stratification risk using the internal sample and TCGA cohort of cases, according to the American Thyroid Association guideline (ATA, 2009). **A and C.** Classification of the cases in three categories: low, intermediate and high risk of relapse. **B and D.** Disease-free survival curves in well-differentiated carcinomas patients stratified into low, intermediate and high risk according to clinical pathological parameters. **Abbreviations.** P: log rank test; WDTC GP: well-differentiated carcinoma with good prognosis; WDTC PP: well-differentiated carcinoma with poor prognosis; *American Thyroid Association (ATA) guidelines (2009).
